# Supplementary figures and images for: EXcellence and PERformance in Track and Field (EXPERT)—A Mixed-Longitudinal Study on Growth, Biological Maturation, Performance, and Health in Young Athletes: Rationale, Design, and Methods (Part 1)
Source: J Funct Morphol Kinesiol. 2026 Jan 1;11(1):25. doi: 10.3390/jfmk11010025 (PMC12821680; doi:10.3390/jfmk11010025)

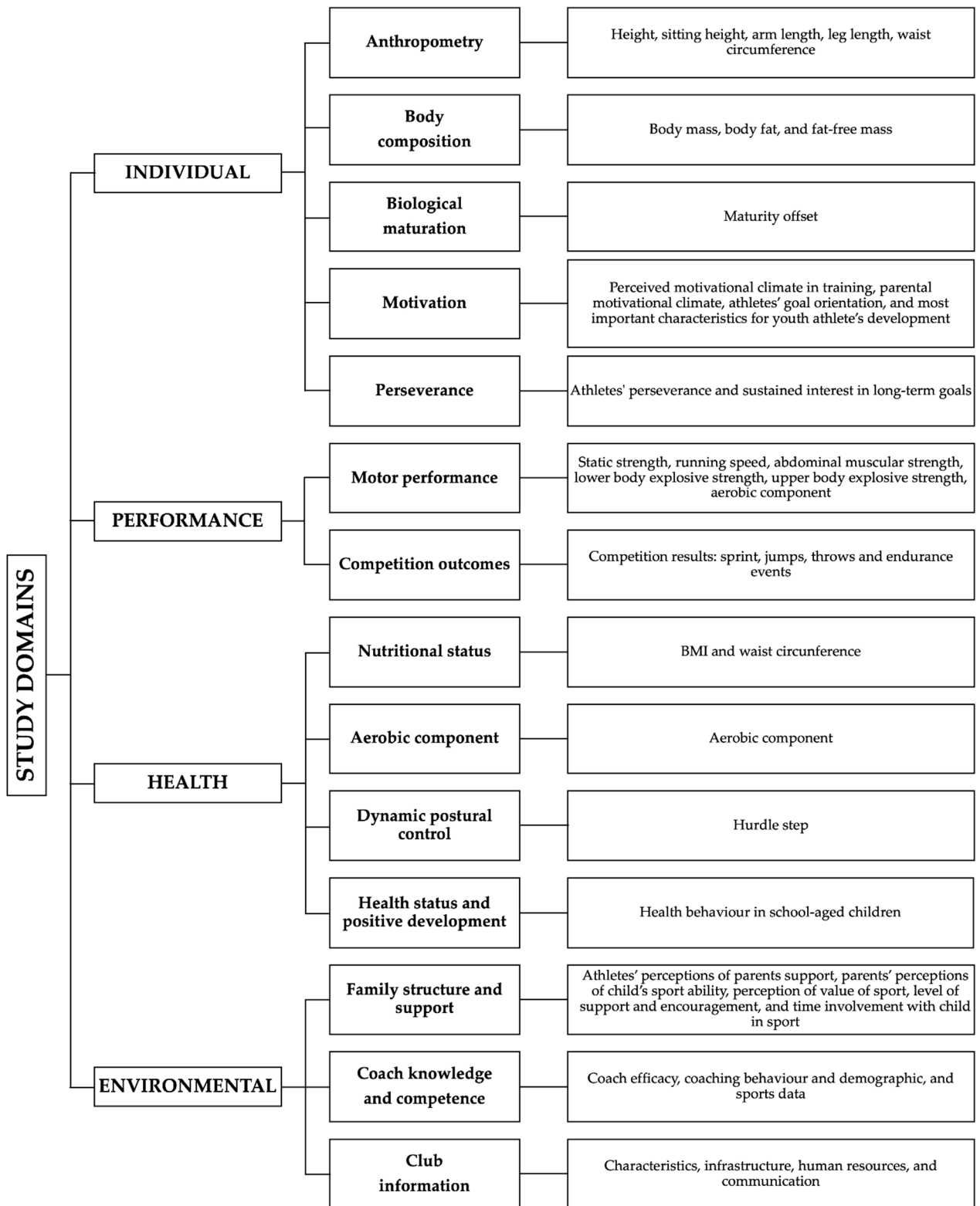

Supplementary file S1: Details of all indicators that will be assessed within each domain.

Supplement: Supplementary file 1 [file jfmk-11-00025-s001.zip › jfmk-3981519-supplementary.pdf]
